# Automated RECOMIA AI-based total metabolic tumor volume in lymphoma – a retrospective study

**DOI:** 10.1186/s13550-026-01403-1

**Published:** 2026-02-27

**Authors:** May Sadik, Johanna Mörk, Jesus Lopez Urdaneta, Malin Lewold, Måns Larsson, Olof Enqvist, Sally F. Barrington, Lars Edenbrandt, Elin Trägårdh

**Affiliations:** 1 Department of Clinical Physiology, Region Västra Götaland, Sahlgrenska University Hospital, Gothenburg, SE-413 45 Sweden; 2 Department of Molecular and Clinical Medicine, Institute of Medicine, Sahlgrenska Academy, University of Gothenburg, Gothenburg, Sweden; 3 Department of Clinical Physiology and Nuclear Medicine, Skåne University Hospital, Malmö, Sweden; 4 Department of Translational Medicine and Wallenberg Center for Molecular Medicine, Lund University, Malmö, Sweden; 5 Eigenvision AB, Lund, Sweden; 6 Department of Electrical Engineering, Chalmers University of Technology, Gothenburg, Sweden; 7 Clinical PET Centre, School of Biomedical Engineering and Imaging Sciences Kings College, London, UK

**Keywords:** FDG PET/CT, Haematological disease, Staging, Convolutional neural networks, Quantification

## Abstract

**Background:**

Increasing evidence suggests that total metabolic tumor volume (tMTV) measured before treatment in lymphoma patients undergoing [18F]fluorodeoxyglucose (FDG) PET/CT scans can predict prognosis. However, there is a lack of fast, reliable, and easy-to-perform multilesional segmentation tools with an urgent need to improve tMTV segmentation workflow in clinical practice. Here, we develop an artificial intelligence (AI)-based tool that automatically calculates tMTV in untreated lymphoma patients undergoing FDG PET/CT. The RECOMIA AI-based tool is a 3D U-Net convolutional neural network trained on a cohort of 1,500 lymphoma patients, mean age 52 years (range 10–88), 44% were female. The model was optimized to segment metabolically active tumors in the FDG PET/CT scans, enabling automated tMTV measurements. The test group consisted of all untreated Hodgkin lymphoma (HL) patients and all Diffuse large B-cell lymphoma (DLBCL) patients who underwent FDG PET/CT at Sahlgrenska University Hospital between 2017–2018 and 2019–2022, respectively. There were 117 patients with mean age 50 years (range 7–90), 39% were female. Nine nuclear medicine physicians manually segmented lesions for tMTV calculations, with each patient independently segmented by two physicians.

**Results:**

The median of the manual tMTV was 321 cm^3^ (interquartile range [IQR]: 92–689 cm^3^) and the median of the difference between two tMTV values segmented by different physicians for the same patient was 26 cm^3^ (IQR: 9–86 cm^3^). In 85 of the 117 patients, one of two manual tMTV measurements was closer to the AI tMTV value than the second manual tMTV measurement made by another physician. In 15 of the remaining 32 patients, the difference between the AI tMTV and the manual tMTV was small (< 26 cm^3^, the median difference between two manual tMTV values made on the same patient).

**Conclusion:**

The results of this study show that the RECOMIA AI-based tool achieved segmentation similarity within the inter-observer variability of experienced nuclear medicine physicians in 85% (100/117) of untreated lymphoma patients. This demonstrates the feasibility of using AI to support physicians in quantifying tMTV for assessment of prognosis in clinical practice.

## Introduction

Increasing evidence indicates that total metabolic tumor volume (tMTV) on baseline [18F]fluorodeoxyglucose (FDG) PET/CT scans predicts prognosis [1]. TMTV has the potential to be particularly valuable imaging biomarker to guide haematologists when selecting new treatment regimens such as immunotherapy [2, 3] since the costly drugs are not affordable for all patients and, are typically reserved for those with the highest probability of benefit.

Currently, it is time-consuming for nuclear medicine physicians and/or radiologists to manually or semi-automatically delineate all pathological lesions in the FDG PET/CT images. Boellaard and coworkers conducted a study by transforming FDG PET/CT-scans to 10 different readers at various sites who used different commercially available software platforms to derive tMTV. The authors concluded that tMTVs can be obtained with reasonable accuracy across readers and platforms, but that the software used should be reproducible, reliable, and fast to be implemented in daily clinical routine [4]. Here, we present an improved artificial intelligence (AI)-based method for automatic tMTV quantification. We tested this method on untreated Hodgkin lymphoma (HL) and Diffuse large B-cell lymphoma (DLBCL) patients undergoing FDG PET/CT, with physicians manually delineating tMTV as gold standard.

## Methods

### Patients

*Test group:* All HL patients from 2017–2018 [5] and all DLBCL patients from 2019–2022 who had staging by FDG PET/CT at Sahlgrenska University Hospital, with biopsy-proven lymphoma were retrospectively included. The patients were newly diagnosed and untreated. The final group consisted of 117 patients with a mean age 50 years (range 7–90), 39% were female. None of these patients were included in the training group.

*Training group:* FDG PET/CT studies from 1,500 patients examined between 2008–2018 at four different hospitals (258 from Sahlgrenska University Hospital, Gothenburg, Sweden, 624 from Skåne University Hospital, Malmö/Lund, Sweden, 503 from The Cancer Imaging Archive (TCIA) from University Hospital Tübingen [6], and 115 from TCIA [7] were used in the training of the RECOMIA AI-based tool. The patients mean age was 52 years (range 10–88), 44% were female. 1,101 patients were diagnosed with lymphoma, and 399 patients with negative scanswho were examined by PET/CT with a clinical indication (e.g. follow-up after tumor resection) but did not show any findings of metabolically active malignant disease. The selection criteria for negative samples were: no detectable FDG-avid tumor lesion according to the clinical radiology report and age >18 years (6). 

The study was approved by the Ethics Committees at Gothenburg and Lund Universities or by the Swedish Ethical Review Authority. The need for written informed consent was waived in #2019–01274 Dnr 2024–08225-02, while all participants provided written informed consent before entering the following studies 2016/417, 2018/117, 2018/753, 2021–05734-02, respectively. We certify that the study was performed in accordance with the ethical standards laid down in the 1964 Declaration of Helsinki and its later amendments. The datasets from anonymized publicly available publications of the TCIA data were approved by the local ethics committee and data protection officer [6, 7].

### Image acquisitions for the test group

FDG PET/CT scans from Sahlgrenska university hospital were obtained using three integrated PET/CT systems. Patients were fasted for at least 6 h prior to administration of FDG and the standard uptake time was 60 min. The adult patients were administered 4 MBq/kg FDG (maximum 400 MBq) with administration for children adjusted according to the EANM Dosage Card (Version 5.7.2016). The field of view for PET scans was from the base of the skull to the mid-thigh, which was matched for accompanying low dose CT scans that were used for attenuation and estimation of scatter correction. On all 3 systems, the CT scan was a 64 slice helical scan acquired at 120 kV and 30 mAs using a 512 × 512 matrix. The CT was reconstructed using a filtered back projection algorithm with a slice thickness and spacing matching those of the PET scan [8, 9].

*Siemens Biograph 64 Truepoint:* PET images were acquired for 3 min per bed position and reconstructed with a slice thickness of 5 mm and slice spacing of 3 mm with an iterative OSEM 3D algorithm (4 iterations and 8 subsets) and a matrix size of 168 × 168.

*GE Healthcare Discovery MI 5R:* PET images were acquired for 2 min per bed position and reconstructed with a slice thickness of 2.8 mm and slice spacing of 1.8 mm with an iterative Bayesian penalized likelihood algorithm (Q.Clear), using a matrix size of 384 × 384.

*GE Healthcare Omni Legend 32:* PET images were acquired for 1 min per bed position and reconstructed with a slice thickness of 2.1 mm and slice spacing of 1.8 mm with an iterative BPL algorithm (Q.Clear) and a medium level deep learning algorithm using a matrix size of 384 × 384.

### Image interpretation

The manual segmentations used to calculate the reference tMTV in the 117 patients in the test group was performed by a team of nine nuclear medicine physicians (SFB, ET, BS, ALN, ALJ, JLL, JLU, RK, ML) from 8 different hospitals. The physicians segmented FDG uptake in tumor sites for tMTV calculations based on the following recommendations [10]:

- Viable areas in lymph nodes with increased FDG uptake
- Focal uptake in the spleen, irrespective of splenic size
- Focal uptake in the bone marrow or other extra-nodal sites
- Diffuse increased uptake in the spleen, in the absence of reactive changes in bone marrow, greater than the liver uptake (spleen/liver ratio >1.5 and bone marrow/liver ratio <1.0)

### RECOMIA-AI-based tool

The automated segmentation of metabolically active tumor regions was performed using a 3D convolutional neural network. The model is based on a four-level U-Net architecture [11], implemented using the MONAI framework [12]. Both training and inference were performed on volumetric image patches of size 192 × 192 × 192 voxels.

The network takes as input a co-registered pair of CT and PET-derived standardized uptake value (SUV) images. Prior to processing, the images were resampled to a uniform voxel size of 2.73 × 2.73 × 2.79 mm. Intensity normalization to the range (0, 1) was applied following clamping of the CT image to the range [–1024, 3072] Hounsfield units and the SUV image to [0, 100]. The output was a segmentation map of the same size as the input images, with two classes: pathological uptake and background.

The model was trained on a dataset of 1,500 FDG PET/CT scans from patients with lymphoma. Of these, 1,200 scans were used for training and 300 for validation. The AI was trained by annotating suspicious hypermetabolic pathological uptake according to the recommendation stated above.The training annotations were collected from multiple readers following international consensus guidelines, reducing bias toward any single reader’s segmentation style.

Training was done for 200 epochs, where one epoch consisted of 10,000 sampled patches. The Nadam optimizer [13] was used with an initial learning rate of 5 × 10⁻^5^, decayed exponentially by a factor of 0.985 after each epoch. The loss function was weighted categorical cross-entropy, assigning a weight of 25.0 to foreground voxels and 1.0 to background voxels. Deep supervision was applied by adding auxiliary loss functions to intermediate layers of the network to improve gradient flow and model convergence [14], with weight of 0.5, 0.25, and 0.125 for intermediate layers.

During training, foreground and background classes were sampled in equal proportions. After every 20 epochs, background sampling was refined based on pixelwise loss values to focus the model on more difficult regions.

Inference was performed using a sliding window approach, with the same patch size as used during training. To reduce edge effects, input patches were overlapped by 94 voxels. For voxels with multiple predictions, the output corresponding to the largest effective receptive field was selected. This method enabled full-volume, patch-wise inference with accurate segmentation boundaries.

Statistical analysis: The Dice score was used to compare the physicians´ tMTV segmentations pairwise with each other and the physicians´ segmentations with the AI-tool. Dice quantify similarity between two sets, with values ranging from 0 (no similarity) to 1 (perfect similarity). 

## Results

**Fig. 1:**
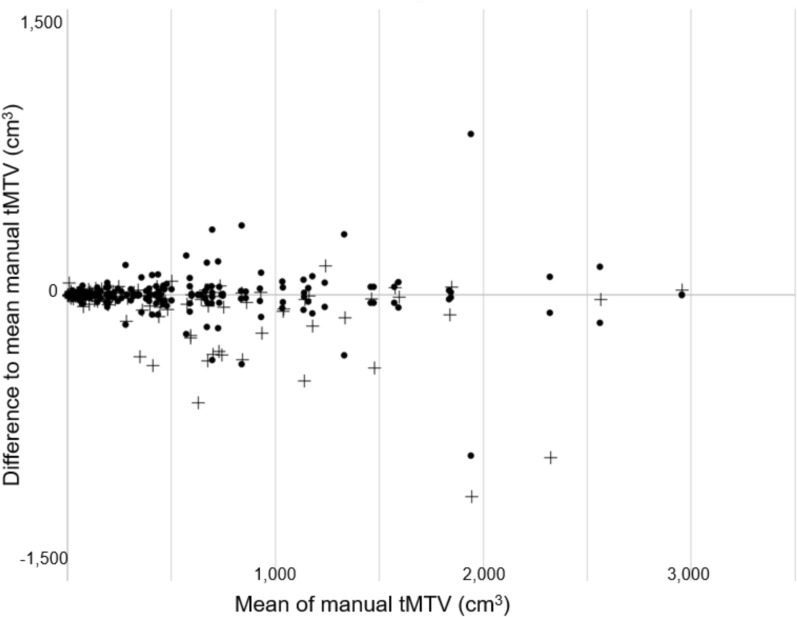
Bland–Altman plot showing the differences between the two manual tMTV values (•), RECOMIA AI-based tMTV ( +) related to the mean of the two manual tMTV values

The median of the manual tMTV was 321 cm^3^ (interquartile range [IQR]: 92–689 cm^3^) and the median of the difference between two tMTV values segmented by different physicians for the same patient was 26 cm^3^ (IQR: 9–86 cm^3^). The median tMTV for the RECOMIA AI-based tool was 272 cm^3^ (IQR: 65–619 cm3). In 85 of the 117 patients, one manual tMTV value was closer to the RECOMIA tMTV value than to the other manual tMTV value segmented by one of the other eight physicians. In 15 of the remaining 32 patients, the difference between the RECOMIA tMTV and the manual tMTV was small (< 26 cm^3^, which was the median difference between two manual tMTV values from the same patient). The RECOMIA AI-based tool could be used without any manual adjustments in 85% (100/117) of the untreated lymphoma patients. The manual and RECOMIA AI-based tMTV values are presented in the Bland–Altman plot, Fig. 1. When the physicians´ segmentations were pairwise compared with each other the average Dice score was 0.77. The corresponding results for physicians´ segmentations compared with the AI-tool was 0.74. Once the PET/CT-images are loaded, the RECOMIA AI-based tool will analyse and present a tMTV-value in less than a minute (median time for analysis 54.8 seconds).

**Fig. 2:**
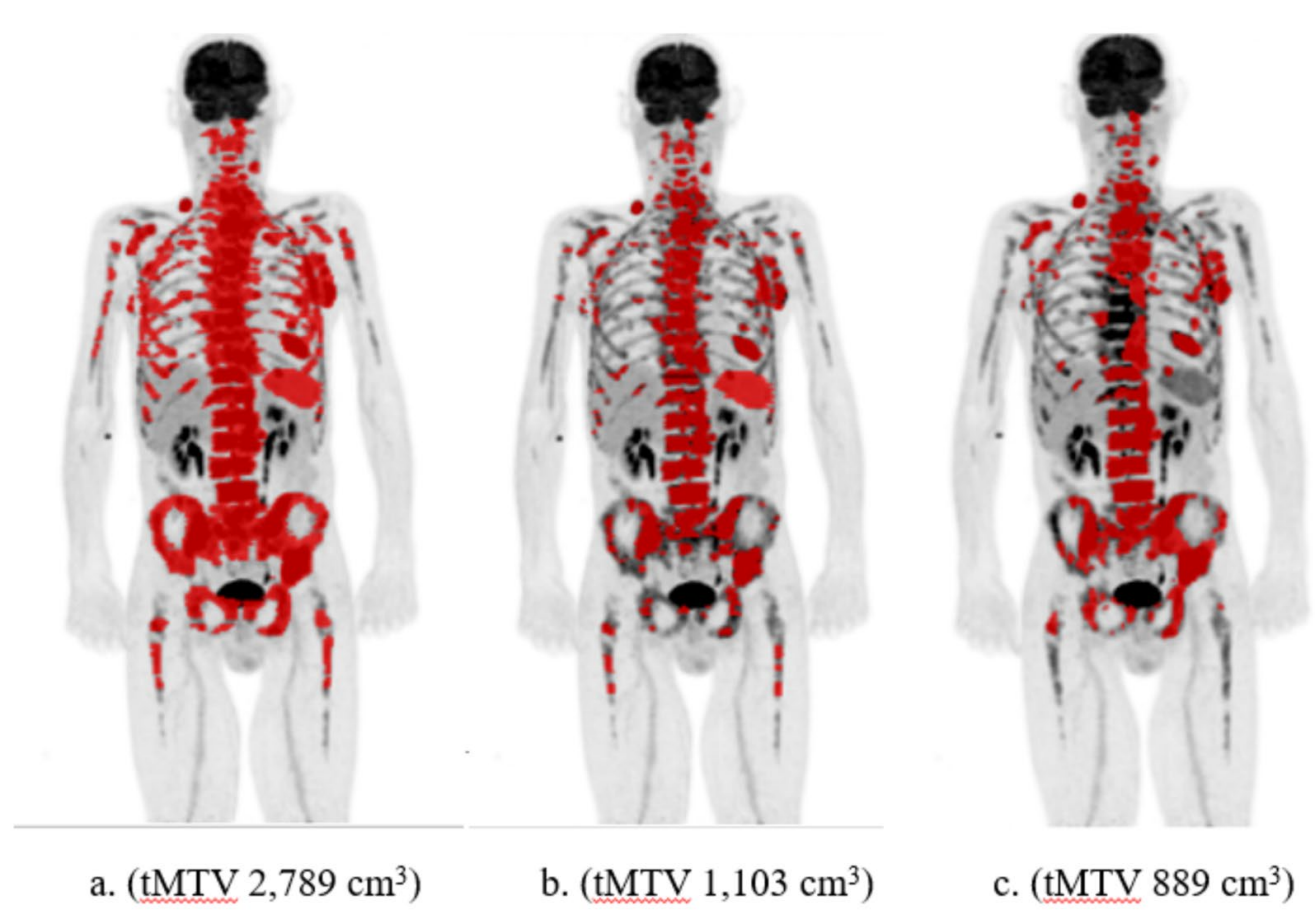
The patient with the largest difference between the mean of the two manual tMTV values (**a** and **b**) and the RECOMIA AI-based tMTV (**c**)

The patient with the largest difference (1,057 cm^3^) between RECOMIA AI-based tMTV (889 cm^3^) and the mean of the two manual tMTV values (2,789 and 1,103 cm^3^) is shown in Fig. 2. The difference between the RECOMIA AI-based and the manual segmentations was mainly due to underestimation of bone involvement. The AI also did not segment the spleen. This was one of the 85 patients in which one of the manual tMTV values (1,103 cm^3^) was closer to the RECOMIA AI-based tMTV value (889 cm^3^) than the other manual tMTV value (2,789 cm^3^).

**Fig. 3:**
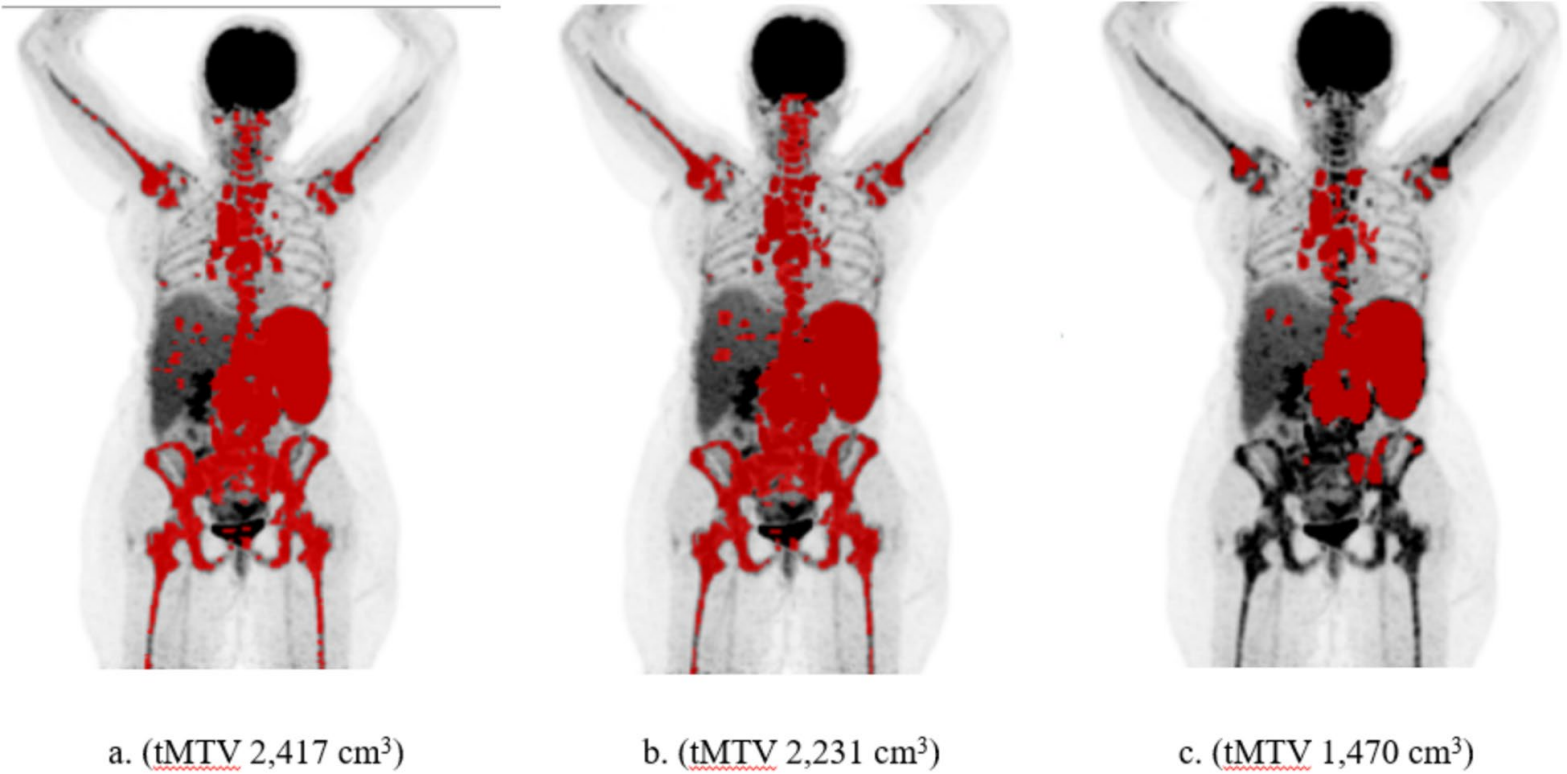
The patient with the second largest difference between RECOMIA AI-based tMTV (**c**) and the two manual tMTV (**a** and **b**) values

Figure 3 shows the patient with the second largest difference between RECOMIA AI-based tMTV (854 cm^3^) and the mean of the two manual tMTV values (2,417 and 2,231 cm^3^). The AI primarily underestimated uptake in bone marrow lesions. The bone uptake was interpreted as widespread focal uptake by the physicians, but most likely as increased diffuse bone marrow activity by the AI.

**Fig. 4:**
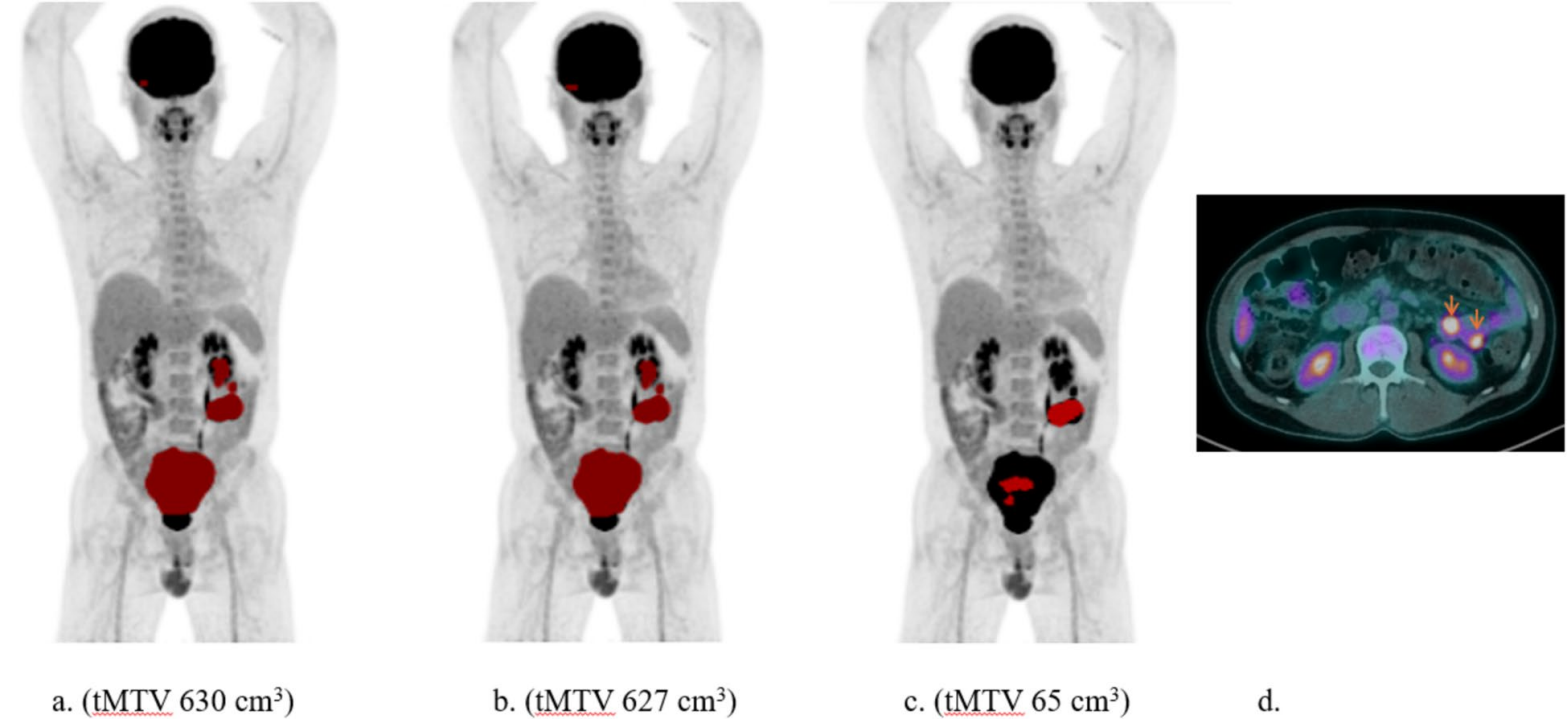
The patient with the third largest difference between the two manual tMTV (**a** and **b**) and the RECOMIA AI-based tMTV (**c**) values. Transverse plane (**d**) with the missed uptake by AI in the bowel in the left lumbar region (arrows)

The patient with the third largest difference between RECOMIA AI-based tMTV (563 cm^3^) and the mean of the two manual tMTV values (630 and 627 cm^3^) is shown in Fig. 4. The pathological uptake in the lower part of the bowel within the pelvis was partly missed by the AI and was mistaken as part of the bladder, and pathological uptake in the bowel in the left lumbar region was missed.

## Discussion

We have demonstrated that the RECOMIA AI-based tool could quantify tMTV accurately in 100 of the 117 (85%) untreated lymphoma patients without any manual adaptation. That was within the range of the inter-reader tMTV or close to the manual tMTV (difference < 26 cm^3^) based on segmentation by nuclear medicine physicians (Fig. 1). The fact that the difference between the two manual tMTV values was greater than the difference between the AI-derived tMTV and one manual tMTV in 73% (85/117) of the patients likely reflects known inter-observer variability in manual tMTV delineation. The variation among readers can arise from differences in lesion interpretation, handling of physiological uptake, and thresholding decisions. This result indicates that the AI tool does not behave as an outlier relative to manual measurements and falls within the range of inter-observer variability between physicians. The Dice score both between the physicians´ segmentations (0.77) and between the physicians´ and the software (0.74) indicate good similarity. This AI-tool is a further development of our earlier version (5). Here, we increased the training group from 101 to 1,500 lymphoma patients from four different hospitals. The goal was to include greater variations and more comprehensive set of pathological locations and distributions as well as a broad range of scanners, acquisition protocols, and patient populations, which was intended to reduce for example scanner-specific bias and enhance the algorithm’s ability to generalize to heterogeneous clinical data. Recent deep-learning–based methods for automated tMTV assessment in lymphoma provide important context for the present work. Yousefirizi et al. introduced TMTV-Net, a fully automated 3D PET/CT segmentation approach based on multiple multi-resolution 3D U-Nets (15). In contrast, the present method employs a more streamlined, end-to-end voxel-wise segmentation approach with reduced architectural complexity. While direct comparison is difficult due to differences in study design and the absence of inter-observer evaluation in their work, the reported segmentation performance of TMTV-Net is comparable to that observed in our study. Other approaches have explored simplified PET representations, such as the application of AI to FDG PET maximum-intensity projections (MIPs) to extract prognostic biomarkers in DLBCL (16). Although computationally efficient, MIP-based methods do not provide explicit 3D tumor delineation and therefore do not directly yield volumetric tMTV. In contrast, our approach performs whole-body voxel-wise segmentation, enabling direct tMTV quantification and visual verification of tumor extent. 

Increasing evidence suggests that imaging biomarkers such as tMTV extracted from PET/CT have prognostic and predictive roles in the selection of lymphoma patients suitable for novel and sometimes costly T cell engaging therapy such as Chimeric Antigen Receptor T-cell (CAR-T). Researchers have reported that patients with baseline low tMTV had significantly superior progression free survival and/or overall survival after CAR-T, however, cut-off values in different studies differed [15, 16] depending on patient population characteristics, number of patients included, and PET/CT-devices used, reflecting that the implementation of tMTV in clinical practice is still in its early stages. We trained our AI by annotating suspicious hypermetabolic pathological uptake in a large patient cohort selected from different hospitals and from publicly available materials using different PET/CT-cameras and manufacturers, i.e. aiming to develop a generalizable tool. The training and testing datasets were annotated following international guidelines. Furthermore, the training of the AI-tool on these 1,500 patients was done by different readers, i.e. the tool is not likely to be biased by one reader´s tMTV segmentation. The test of the software included nine nuclear medicine physicians from different hospitals and the 117 cases were randomly distributed to each of them, i.e. the calculation of individual physician’s observer variations is not feasible. It is well-established that image segmentation is associated with inter-observer variability, and that using AI could significantly increase agreement between physicians. 

For such tools to be implemented in the clinical routine they should need no or minimal manual adjustments and should be reproducible, reliable, and fast. Once the PET/CT-images are loaded, the RECOMIA AI-based tool calculates and displays a tMTV-value with median analysis time of 55 seconds. No tMTV-comparison was made with other existing tools because no alternative software was clinically available at Sahlgrenska University hospital, therefore the results in the present study cannot be directly related to others. Boellaard and co-workers on the other hand transferred PET/CT-scans to 10 readers who used different commercially available software platforms at different sites to derive tMTV. There results showed that tMTVs could be obtained with reasonable accuracy across readers and platforms (within 10% compared with reference benchmark values for most tMTVs) but that processing times could vary considerably depending on reader experience and the software platform (4). The first step in the development and validation of our RECOMIA AI-based tool was to test it on our own consecutive patient group were all HL and DLBCL patients examined during a certain time interval were included. The second step will be to apply the tool to the benchmark dataset of 60 lymphoma patients with baseline FDG PET/CT tMTV assessment, which is agreed upon by an international panel of experts.

The two cases with the largest differences in tMTV between the RECOMIA AI-based tool and manual segmentations were mainly due to underestimation of the pathological uptake in bone (Fig. 2 and 3). An explanation could be that the training group lacked cases with such a widespread bone marrow involvement. A large difference in tMTV between AI and physicians is not necessarily a clinical problem, since patients with extensive disease may not be candidates for immunotherapy [17- 27].

The case with the third largest difference between RECOMIA AI-based tMTV and the manual is due to underestimation from the tool because the AI misclassified pathological uptake as physiological uptake in the bladder and bowel. More such cases must be included in the training in future refinements of the tool.

The AI model is freely available for research use on the RECOMIA platform (www.recomia.org).

## Conclusion

The results of this study show that the RECOMIA AI-based tool achieved segmentation similarity within the inter-observer variability of experienced nuclear medicine physicians in 85% (100/117) of the untreated lymphoma patients. This demonstrates the feasibility of using AI to support physicians in quantifying tMTV for assessment of prognosis in clinical practice.

## Data Availability

The datasets generated and/or analysed during the current study are not publicly available due to ethical considerations.

## Abbreviations

AI: Artificial intelligence
BPL: Bayesian penalized likelihood
3D: 3 Dimensional
DLBCL: Diffuse large B-cell lymphoma
FDG PET/CT: [18F] fluorodeoxyglucose positron emission tomography/computed tomography
HL: Hodgkin lymphoma
IQR: Interquartile range
MIPs: Maximum-intensity projections SUV: Standardized uptake value
TCIA: The cancer imaging archive
tMTV: Total metabolic tumor volume
